# FAAH Inhibition Reverses Depressive-like Behavior and Sex-Specific Neuroinflammatory Alterations Induced by Early Life Stress

**DOI:** 10.3390/cells13221881

**Published:** 2024-11-14

**Authors:** Anna Portugalov, Irit Akirav

**Affiliations:** 1 School of Psychological Sciences, Department of Psychology, University of Haifa, Haifa 3498838, Israel; anuta89.ap@gmail.com; 2 The Integrated Brain and Behavior Research Center (IBBRC), University of Haifa, Haifa 3498838, Israel

**Keywords:** cannabinoids, depression, early life stress, neuroinflammation, URB597

## Abstract

Early life stress (ELS) increases predisposition to major depressive disorder (MDD), with neuroinflammation playing a crucial role. This study investigated the long-term effects of the fatty acid amide hydrolase (FAAH) inhibitor URB597 on ELS-induced depressive-like behavior and messenger RNA (mRNA) of pro-inflammatory cytokines in the medial prefrontal cortex (mPFC) and CA1 regions. We also assessed whether these gene expression alterations were present at the onset of URB597 treatment during late adolescence. ELS induced a depressive-like phenotype in adult male and female rats, which was reversed by URB597. In the mPFC, ELS downregulated nuclear factor kappa B1 (nfκb1) in both sexes, while URB597 normalized this expression exclusively in males. In females, ELS downregulated interleukin (il) 6 and tumor necrosis factor alpha (tnfα) but upregulated il1β and corticotropin-releasing factor (crf); URB597 normalized il6, il1β, and crf. In the CA1, ELS downregulated il1β and tnfα in males and upregulated il1β expression in females, which was reversed by URB597. Some of these effects began in late adolescence, including mPFC-nfκb1 expression in both sexes, mPFC-il6 and mPFC-il1β in females, CA1-il1β and CA1-tnfα in males, and CA1-il1β in females. These findings highlight URB597 as a therapeutic approach for reversing ELS-induced depressive-like behavior by associating with changes in the gene expression of neuroinflammatory cytokines, with notable sex differences.

## 1. Introduction

Major depressive disorder (MDD) is a complex debilitating psychological disorder that cannot be fully explained by any single established biological or environmental pathway [1]. Extensive research has shown that early life stress (ELS) markedly increases the risk of developing severe forms of MDD later in life, which often do not respond well to conventional antidepressant treatments [2,3,4]. Childhood abuse and parental neglect can lead to heightened inflammatory responses in adulthood and affect neuroendocrine stress responses by altering the function of the hypothalamic–pituitary–adrenal (HPA) axis [5,6,7,8]. For example, mice exposed to maternal deprivation exhibit increased corticotropin-releasing factor (CRF) messenger RNA (mRNA) expression in the paraventricular nucleus (PVN), resulting in elevated corticosterone levels [9]. ELS has also been shown to heighten susceptibility to neuropsychiatric disorders via epigenetic mechanisms, including DNA methylation, histone modifications, noncoding RNA activity, and altered neuronal signaling [10,11]. These changes are linked to impairments in memory and learning processes [12,13].

ELS can activate microglia, promoting the transcription of pro-inflammatory cytokines, such as nuclear factor kappa B (NF-κB), interleukins (IL), IL-1β,IL-6, and tumor necrosis factor-alpha (TNF-α) [14]. ELS-induced alterations in pro-inflammatory cytokine levels may play a crucial role in developing dysfunctions associated with HPA axis function, such as MDD [15]. Growing evidence suggests that IL-1β,IL-6, TNF-α, and NF-κB are central to the association between ELS and increased risk for MDD [16,17,18].

Considering the critical role of neuroinflammation in the onset of MDD, targeting neuroinflammatory pathways offers a promising therapeutic strategy for treating this disorder [19]. The endocannabinoid system (ECS) is known to have a significant role in regulating neuroinflammatory responses [20,21]. Recent studies highlight the anti-inflammatory properties of URB597 (URB), a fatty acid amide hydrolase (FAAH) inhibitor [22,23,24]. URB acts as a highly potent and selective FAAH inhibitor, which terminates anandamide (AEA) signaling in the synapse [25,26]. Treatment with URB (0.3 mg/kg, intraperitoneally (i.p.), for twelve weeks) improved neuroinflammatory responses in a chronic cerebral hypoperfusion model by inhibiting NF-κB pathways and lowering IL-1β and TNF-α levels [22]. In another study, URB administration (0.3 mg/kg, i.p.), followed by oral ethanol, reduced ethanol-induced increases in TNF-α levels in the medial prefrontal cortex (mPFC) and hippocampus [23]. 

Our prior research showed that ELS leads to depressive-like behaviors and alters microRNAs (miR) expression in the mPFC in a sex-dependent manner [27]. Specifically, male and female rats were exposed to the “Limited Bedding and Nesting (LBN)” paradigm, which disrupts maternal care by placing the mother in conditions insufficient for adequate pup care [28]. During the late adolescent period, the rats were treated with either vehicle or URB (0.4 mg/kg, i.p. for two weeks). In adulthood, ELS-exposed males and females exhibited hypo-locomotion, impaired sociability and social short-term memory, and increased learned helplessness; late adolescent URB treatment reversed these behaviors. Interestingly, ELS reduced miR-16 in the mPFC of males and miR-135a in the mPFC of females, with URB treatment restoring these levels. Additionally, in females, ELS resulted in an upregulation of CB2 receptor expression and a reduction in FAAH gene expression, changes that were also reversed by URB treatment. These results indicate that while ELS induces similar behavioral phenotypes in both sexes, it causes different molecular alterations in a sex-dependent manner.

This study aimed to examine the antidepressant effects of URB597 on behavior and inflammatory gene expression (nfκb1, il1β, il6 and thfα) in the mPFC and hippocampal CA1 region of adult male and female rats exposed to ELS. Additionally, we explored the impact of ELS and URB597 treatment on stress-related gene expression (glucocorticoid receptor nr3c1 and crf) in the mPFC, as the NF-κB pathway can be modulated by glucocorticoids [29]. Finally, we aimed to examine whether the gene alterations observed in adulthood were present earlier, at the onset of URB treatment during late adolescence. 

## 2. Materials and Methods

### 2.1. Subjects

Eight pregnant Sprague Dawley (SD) dams (Envigo, Jerusalem, Israel) were housed in polypropylene cages (59 × 28 × 20 cm^3^). After parturition, pups were weaned on postnatal day (P)21 and randomly assigned to one of four groups (*n* = 5 per group) by sex: NoELS-Vehicle, NoELS-URB, ELS-Vehicle, and ELS-URB. Group sizes were determined based on our previous research [27]. Pups were kept at 22 ± 2 °C in a 12 h light/dark cycle (lights on at 07:00) with unlimited access to food and water. To ensure each treatment group was equally represented, no more than one male and one female from each litter were randomly assigned to each experimental condition. The University of Haifa Ethics and Animal Care Committee approved all procedures, with steps taken to minimize pain and discomfort (approval numbers: 347U and 3089U).

### 2.2. Early Life Stress (ELS) Model 

For the ELS model, we employed the Limited Bedding and Nesting (LBN) paradigm [30,31,32] with slight modifications [27,33]. From postnatal days (P)7 to P14, the ELS dams and their pups were kept in cages containing a limited amount of “Sunny Chips” bedding material (1.2 cm layer). In contrast, the NoELS dams and their pups were kept in cages with abundant bedding material (7–9 cm layer).

### 2.3. Pharmacological Treatment

URB597 [0.4 mg/kg; i.p.; Cayman chemicals, Ann Arbor, MI, USA; [27]] was prepared by dissolving it in dimethyl sulfoxide (DMSO), saline (0.9% NaCl), and Tween 80, ensuring the final DMSO concentration was below 2%. Control groups received injections of the vehicle solution (DMSO, saline-0.9% NaCl, and Tween 80, also maintaining a final DMSO concentration of less than <2%). Injections were administered daily over a 15-day period, with the dosage and timing during late adolescence (P45-60) determined based on previous studies [27,34].

### 2.4. Behavioral Tests

Behavioral tests were performed 30 days after the final injection to assess the long-term effects of URB597. Males and females were tested separately, following a specific sequence of tests: activity in a novel open-field arena (OFT), sociability (social preference—SP) and short-term social memory (social recognition—SR), and the forced swim test (FST). The social tests (SP and SR) were conducted in the OFT arena after a three-day habituation period. The most stressful test, the FST, was performed last. All animals completed the tests in a consistent sequence. Testing was conducted under dim lighting conditions (15–20 lx) between 07:00 a.m. and 1:00 p.m., during the light phase. The number of animals varied across tests, as some were excluded due to having standard deviations that significantly differed from the group. Specifically, any rat with a Z-score exceeding 2.5 standard deviations from the mean was excluded. Fewer rats were used for biochemical analyses than for behavioral tests.

#### 2.4.1. Activity in an Open-Field Test (OFT)

The arena is a square black open field measuring 50 × 50 × 50 cm^3^, with the floor divided into 25 squares, each 10 × 10 cm^2^, outlined by 1 cm wide white lines. The arena was thoroughly cleaned with 70% ethanol between each animal’s test. Rat movements were recorded and analyzed for 30 min using a video tracking system (Ethovision ×T 14.0, Noldus Information Technology, NBT Ltd., Jerusalem, Israel) to assess locomotion, which was quantified as the distance traveled in centimeters. 

#### 2.4.2. Social Preference (SP) and Social Recognition (SR)

This task is designed to evaluate sociability and short-term social memory. A novel juvenile rat was placed in a separate section of the open field (50 × 50 × 50 cm^3^) using a transparent perforated Plexiglas panel (corrals) [27]. The first phase involved social preference (SP), where the experimental rat had 5 min to explore both a novel juvenile rat and a novel object. After a 30 min interval in a holding cage, the social recognition (SR) phase was performed. During this phase, the rat had 5 min to explore both a familiar juvenile and a novel juvenile, which were also confined within the corrals. The trials were recorded using Dericam, Indoor Pan/tilt IP camera M801W (ShenZhen, China). An exploration index was calculated: for SP, it was the time spent exploring the novel juvenile rat divided by the total exploration time (object + juvenile rat); for SR, it was the time spent exploring the novel juvenile rat divided by the total exploration time (familiar + novel juvenile).

#### 2.4.3. Forced Swim Test (FST)

This test evaluates learned helplessness using a cylindrical water container with a diameter of 62 cm and a height of 40 cm, filled with water and maintained at 23 °C. The water level was adjusted so that the rat could not reach the bottom with its hind paws. Two swimming sessions were conducted: a 15 min pretest (learning phase) followed by a 5 min test the next day. Video recordings from each session of the FST were analyzed to distinguish between passive coping (immobility) and active coping (swimming) strategies [35].

#### 2.4.4. Real-Time (RT) PCR

Rats were euthanized, and brain tissues from the mPFC (prelimbic and infralimbic cortex) and dorsal CA1 region were harvested for molecular analysis [see Figure S1 in the Supplementary Information (SI)]. RNA extraction, cDNA preparation, and quantitative real-time (qRT)-PCR were performed as previously outlined [27,36] to assess mRNA expression of inflammatory genes (nfκb1, il1β, il6, and tnfα ) and stress-related genes (nr3c1 and crf). A total of 1000 ng RNA was converted into cDNA using the qScript cDNA Synthesis Kit (Quanta Biosciences, Gaithersburg, MD, USA). Real-time SYBR Green qRT-PCR amplification was conducted with specific primers (Quanta Biosciences, Gaithersburg, MD, USA) following the manufacturer’s protocols. RT reactions were executed on a Step One real-time PCR system (Applied Biosystems, Waltham, MA, USA). Fold-change values were calculated using the ddCt method relative to the housekeeping gene hypoxanthine phosphoribosyl transferase (HPRT). Primers (see Table S1) were designed and synthesized by Agentek (Tel Aviv, Israel). The suitability of the primers was evaluated through standard curve analysis, melting curve analysis, and an assessment of linearity and threshold [37].

#### 2.4.5. Statistical Analysis

The results are expressed as means ± SEM. The statistical analysis performed included one-way ANOVA, two-way ANOVA, three-way ANOVA, two-way ANOVA with a log link function, three-way ANOVA with a log link function, one-sample *t*-test, independent-samples *t*-test, and Pearson bivariate correlation test, as appropriate. Post hoc comparisons were carried out using independent-samples *t*-test. A significance level of *p* ≤ 0.05 was established. Given previous findings demonstrating sex differences in stress responses, behavior, the neuroendocrine system, gene methylation, and the pathophysiology of MDD [33,34,38,39], separate statistical analyses were conducted for each sex. Data were analyzed using SPSS 27 (IBM, Chicago, IL, USA) and Jamovi 2.3.18 (Sydney, Australia). Normality assumptions were assessed using the Kolmogorov–Smirnov and Shapiro–Wilk tests.

## 3. Results

### 3.1. Experiment 1: Chronic Late Adolescence URB597 Treatment Ameliorates ELS-Induced Depressive-like Phenotype and Modulates Changes in Gene Expression in Adult Male and Female Rats

#### 3.1.1. Chronic Late Adolescence URB597 Treatment Ameliorates ELS-Induced Depressive-like Phenotype in Adult Males and Females

**Figure 1:**
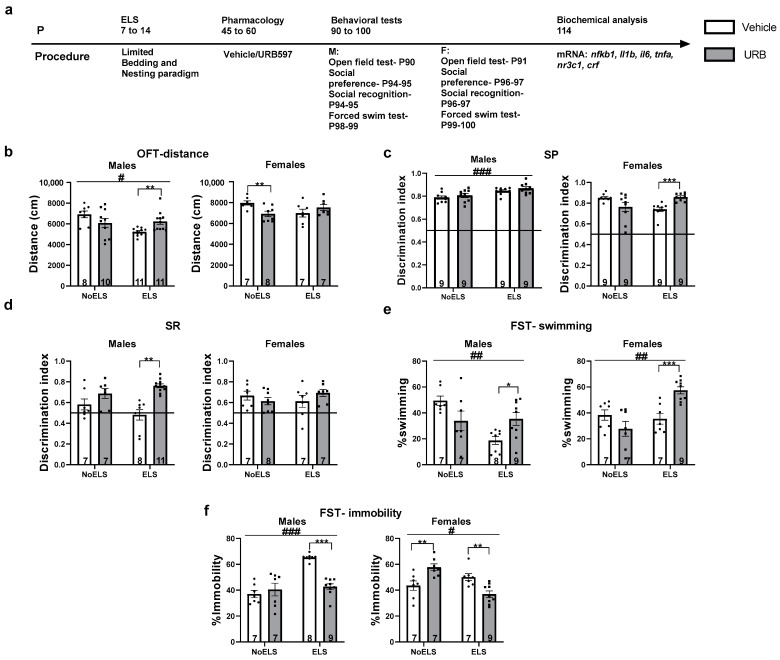
Chronic treatment with URB597 during late adolescence ameliorates the depressive-like phenotype induced by early life stress (ELS) in adult males and females. (**a**) Male and female rats were subjected to ELS from postnatal day (P)7 to P14. During late adolescence (P45-60), they received either a vehicle or URB597 injection. Behavioral assessment commenced on P90, and the rats were euthanized on P114 for molecular analysis (n = 7–11 for all groups). (**b**) In the open-field test (OFT), ELS resulted in reduced distance traveled by males, while URB treatment significantly increased this distance compared to vehicle-treated controls (**left**). In females (**right**), URB treatment led to a decrease in distance traveled in NoELS females compared to vehicle-treated females. (**c**) In the Social Preference (SP) test, ELS increased SP in males compared to NoELS males (**left**). In ELS females (**right**), treatment with URB enhanced SP compared to treatment with vehicle. (**d**) In the Social Recognition (SR) test, treatment with URB led to increased SR in ELS males compared to treatment with vehicle (**left**). No significant differences were found in females (**right**). (**e**) In the Forced Swim Test (FST), ELS decreased the swimming ratio in both sexes compared to NoELS groups. URB treatment increased the swimming ratio in ELS rats compared to treatment with vehicle. (**f**) In the FST, ELS increased the immobility ratio in males (**left**) compared to NoELS groups. URB treatment in ELS males decreased the immobility ratio compared to treatment with vehicle. In females (**right**), treatment with URB led to increased immobility ratio in NoELS rats compared to treatment with vehicle, whereas treatment with URB decreased the immobility ratio compared to vehicle treatment. mRNA: messenger RNA. *, *p* < 0.05; **, *p* < 0.01; ***, *p* < 0.001 indicate statistically significant effects followed by post hoc comparisons; #, *p* < 0.05; ##, *p* < 0.01; ###, *p* < 0.001 indicate statistical significance in main effects.

Male and female rats subjected to ELS received chronic treatment with URB597 during late adolescence, and their depressive-like behaviors were evaluated in adulthood (see Figure 1a for experimental design, with n = 7–11 per sex per group). A three-way ANOVA (2 × 2 × 2) was conducted, considering sex, ELS, and drug as factors. When significant effects related to sex or three-way interactions were observed, data were analyzed separately by sex. For descriptive statistics, see SI Table S2. 

##### Distance Traveled in the Open Field (OFT)

A three-way ANOVA revealed significant effects of sex (F(1,61) = 31.8, *p* < 0.001), ELS (F(1,61) = 4.63, *p* = 0.035), and the interaction between ELS and drug treatment (F(1,61) = 15.56, *p* < 0.001). However, there were no significant effects for drug treatment (F(1,61) = 0.15, *p* = 0.701), the interaction between ELS and sex (F(1,61) = 1.78, *p* = 0.18), the interaction between sex and drug (F(1,61) = 0.62, *p* = 0.43), or the three-way interaction among ELS, drug, and sex (F(1,61) = 0.08, *p* = 0.77).

In males (Figure 1b, left), a two-way ANOVA revealed significant effects of ELS (F(1,36) = 6.09, *p* = 0.018) and a significant interaction between ELS and drug treatment (F(1,36) = 8.94, *p* = 0.005), while there was no significant effect of drug treatment alone (F(1,36) = 0.08, *p* = 0.77). Males exposed to ELS traveled a shorter distance than the NoELS group, indicating ELS-induced hypoactivity. An independent-samples *t*-test showed that ELS-URB males traveled a greater distance compared to those treated with vehicle (t(12.955) = −3.316, *p* = 0.006), suggesting that URB effectively restored activity levels diminished by ELS.

In females (Figure 1b, right), a two-way ANOVA revealed a significant interaction between ELS and drug treatment (F(1,25) = 7.84, *p* = 0.01), with no significant main effects for ELS (F(1,25) = 0.389, *p* = 0.53) or drug treatment (F(1,25) = 0.811, *p* = 0.37). An independent-samples *t*-test indicated that No-ELS-Vehicle females traveled a greater distance compared to those treated with URB (t(13) = 3.138, *p* = 0.008), suggesting that URB influenced activity levels in non-stressed females.

##### Social Preference Test (SP)

A three-way ANOVA indicated significant interactions among ELS × sex (F(1,64) = 4.97, *p* = 0.029), ELS × drug (F(1,64) = 12.11, *p* = 0.001), and ELS × drug × sex (F(1,64) = 11.26, *p* = 0.001). However, there were no significant main effects for sex (F(1,64) = 2.32, *p* = 0.13), ELS (F(1,64) = 2.905, *p* = 0.093), drug (F(1,64) = 1.32, *p* = 0.253), or the interaction between sex and drug (F(1,64) = 0.034, *p* = 0.85).

In males (Figure 1c, left), a two-way ANOVA revealed a significant effect of ELS (F(1,32) = 15.87, *p* < 0.001). However, there were no significant effects for the drug (F(1,32) = 1.83, *p* = 0.185) or the ELS × drug interaction (F(1,32) = 0.016, *p* = 0.901). Males exposed to ELS exhibited a greater social preference compared to their NoELS counterparts. Notably, all groups demonstrated a significant difference from the chance level (0.5 DI), indicating that social preference was preserved across all male groups: NoELS-Vehicle: t(8) = 20.077, *p* < 0.001; NoELS-URB: t(8) = 16.752, *p* < 0.001; ELS-Vehicle: t(8) = 32.985, *p* < 0.001; ELS-URB: t(8) = 24.039, *p* < 0.001.

In females (Figure 1c, right), a two-way ANOVA revealed a significant ELS × drug interaction effect (F(1,32) = 15.45, *p* < 0.001), with no significant main effects of ELS (F(1,32) = 0.092, *p* = 0.764) or drug (F(1,32) = 0.31, *p* = 0.58). Independent-samples *t*-tests indicated that ELS-URB females displayed a significantly higher social preference than ELS-Vehicle females (t(16) = 4.832, *p* < 0.001). Consistent with the male findings, all female groups demonstrated a significant difference from the 0.5 threshold, indicating intact social preference across groups [NoELS-Vehicle: t(8) = 31.131, *p* < 0.001; NoELS-URB: t(8) = 5.864, *p* < 0.001; ELS-Vehicle: t(8) = 11.503, *p* < 0.001; ELS-URB: t(8) = 29.127, *p* < 0.001].

##### Social Recognition Test (SR)

A three-way ANOVA showed significant main effects of drug (F(1,54) = 12.084, *p* = 0.001), as well as significant interactions for sex × drug (F(1,54) = 9.052, *p* = 0.004) and ELS × drug (F(1,54) = 6.706, *p* = 0.012). No significant effects were found for sex (F(1,54) = 0.37, *p* = 0.544), ELS (F(1,54) = 0, *p* = 0.99), ELS × sex interaction (F(1,54) = 0.184, *p* = 0.669), or the ELS × drug × sex interaction (F(1,54) = 0.122, *p* = 0.728).

In males (Figure 1d, left), two-way ANOVA revealed significant main effects of drug (F(1,29) = 22.15, *p* < 0.001) and an ELS × drug interaction (F(1,29) = 4.55, *p* = 0.041), with no significant main effect of ELS (F(1,29) = 0.09, *p* = 0.756). An independent-samples *t*-test indicated that ELS-URB males demonstrated greater social recognition compared to ELS-Vehicle males (t(8.945) = 5.233, *p* = 0.001). Notably, a significant difference from the 0.5 DI threshold was observed only in the NoELS-URB and ELS-URB groups, indicating intact social recognition among URB-treated males [NoELS-Vehicle: t(6) = 1.55, *p* = 0.17; NoELS-URB: t(6) = 3.974, *p* = 0.007; ELS-Vehicle: t(7) = −0.372, *p* = 0.72; ELS-URB: t(10) = 14.03, *p* < 0.001].

In females (Figure 1d, right), two-way ANOVA showed no significant main effects of ELS (F(1,25) = 0.087, *p* = 0.771), drug (F(1,25) = 0.104, *p* = 0.75), or an ELS × drug interaction (F(1,25) = 2.38, *p* = 0.135). However, a significant difference from the 0.5 DI threshold was observed in the NoELS groups and ELS-URB females, suggesting intact social recognition in these groups but impaired social recognition in ELS-Vehicle females [NoELS-Vehicle: t(6) = 3.905, *p* = 0.008; NoELS-URB: t(7) = 3.208, *p* = 0.015; ELS-Vehicle: t(6) = 1.942, *p* = 0.1; ELS-URB: t(6) = 5.576, *p* = 0.001].

##### Swimming in the Forced Swim Test (FST)

A three-way ANOVA revealed significant interactions for ELS × sex (F(1,53) = 18.36, *p* < 0.001) and ELS × drug (F(1,53) = 24.51, *p* < 0.001), with no significant main effects for sex (F(1,53) = 2.72, *p* = 0.105), ELS (F(1,53) = 0.034, *p* = 0.855), or drug (F(1,53) = 0.91, *p* = 0.343). Additionally, no significant interactions were found for sex × drug (F(1,53) = 0.625, *p* = 0.433) or the ELS × drug × sex interaction (F(1,53) = 0.001, *p* = 0.982).

In males (Figure 1e, left), a two-way ANOVA revealed significant main effects of ELS (F(1,27) = 8.48, *p* = 0.007) and an ELS × drug interaction (F(1,27) = 10.32, *p* = 0.003), with no significant effect of drug (F(1,27) = 0.012, *p* = 0.915). ELS males exhibited shorter swim durations compared to NoELS males, indicating a reduction in active coping due to ELS. An independent-samples *t*-test showed that ELS-URB males swam longer than ELS-Vehicle males (t(15) = 2.799, *p* = 0.014), suggesting that URB treatment reversed the ELS-induced reduction in active coping.

In females (Figure 1e, right), a two-way ANOVA also revealed significant effects of ELS (F(1,26) = 10.39, *p* = 0.003) and an ELS × drug interaction (F(1,26) = 15.28, *p* = 0.001), with no significant main effect of drug (F(1,26) = 1.88, *p* = 0.181). An independent-samples *t*-test indicated that ELS-URB females swam for a longer duration compared to ELS-Vehicle females (t(14) = 4.59, *p* < 0.001), suggesting that URB also restored active coping in ELS females.

##### Immobility in the FST

A three-way ANOVA revealed significant main effects of drug (F(1,53) = 4.83, *p* = 0.032) and interactions for ELS × sex (F(1,53) = 29.76, *p* < 0.001), ELS × drug (F(1,53) = 42.46, *p* < 0.001), and sex × drug (F(1,53) = 6.01, *p* = 0.018). No significant effects were found for sex (F(1,53) = 0.11, *p* = 0.734), ELS (F(1,53) = 3.93, *p* = 0.052), or the three-way ELS × drug × sex interaction (F(1,53) = 0.022, *p* = 0.882).

In males (Figure 1f, left), a two-way ANOVA showed significant main effects of ELS (F(1,27) = 27.33, *p* < 0.001) and drug (F(1,27) = 10.68, *p* = 0.003), as well as a significant ELS × drug interaction (F(1,27) = 20.01, *p* < 0.001). ELS males displayed increased immobility compared to NoELS males, indicating ELS-induced learned helplessness. An independent-samples *t*-test showed that ELS-URB males exhibited reduced immobility compared to ELS-Vehicle males (t(15) = −8.975, *p* < 0.001), suggesting that URB reversed the ELS-induced learned helplessness.

In females (Figure 1f, right), a two-way ANOVA revealed significant effects of ELS (F(1,26) = 6.11, *p* = 0.02) and an ELS × drug interaction (F(1,26) = 22.54, *p* < 0.001), with no significant main effect of drug (F(1,26) = 0.033, *p* = 0.858). An independent-samples *t*-test showed that ELS-URB females exhibited reduced immobility compared to ELS-Vehicle females (t(14) = −3.569, *p* = 0.003). Conversely, NoELS-URB females displayed increased immobility compared to NoELS-Vehicle females (t(12) = 3.156, *p* = 0.008), indicating a differential effect of URB in stressed versus non-stressed females.

No significant differences in climbing behavior were observed between the groups in both sexes (see SI Figure S3).

#### 3.1.2. Chronic Late Adolescence URB597 Treatment Ameliorates ELS-Induced Changes in Inflammatory Gene Expression in the mPFC

##### nfκb1

A three-way ANOVA revealed significant main effects of sex (F(1,74) = 195.17, *p* < 0.001) and ELS (F(1,74) = 3.96, *p* = 0.05), as well as a significant ELS × drug × sex interaction (F(1,74) = 12.21, *p* = 0.001). No significant effects were found for drug (F(1,74) = 2.49, *p* = 0.118), ELS × sex interaction (F(1,74) = 1.75, *p* = 0.19), sex × drug interaction (F(1,74) = 0.04, *p* = 0.841), or ELS × drug interaction (F(1,74) = 2.69, *p* = 0.105).

**Figure 2:**
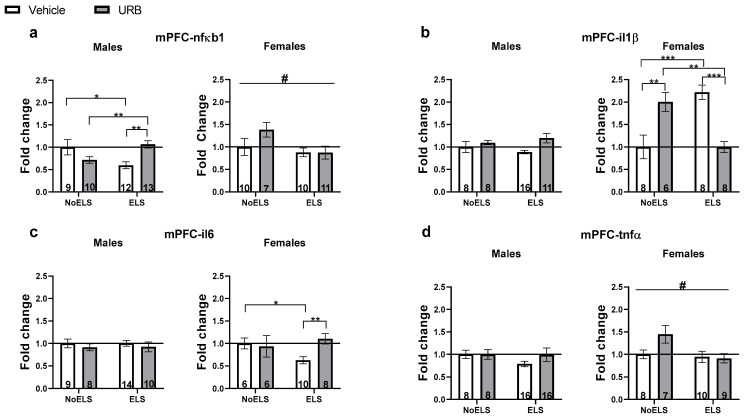
Chronic URB597 treatment during late adolescence ameliorates ELS-induced alterations in the expression of inflammatory genes in the mPFC. (**a**) In males (**left**), URB treatment normalized ELS-induced downregulation of nfκb1 and reduced nfκb1 expression in the NoELS group. In females (**right**), ELS downregulated nfκb1 expression. (**b**) In males (**left**), no significant differences were found in il1β expression. In females (**right**), URB treatment normalized ELS-induced il1β upregulation. Additionally, in the NoELS group, URB increased il1β expression compared to both the NoELS-Vehicle and ELS-URB groups. (**c**) In males (**left**), no significant differences were found in il6 expression. In females (**right**), URB treatment normalized the ELS-induced il6 downregulation. (**d**) In males (**left**), no significant differences were found in the expression of tnfα in males. In females (**right**), ELS reduced tnfα expression. ELS: early life stress; mPFC: medial prefrontal cortex; URB: URB597. *, *p* < 0.05; **, *p* < 0.01; ***, *p* < 0.001 indicate statistically significant effects followed by post hoc comparisons; #, *p* < 0.05 indicate statistical significance in main effects.

In males (Figure 2a, left), a two-way ANOVA revealed a significant ELS × drug interaction (F(1,40) = 15.28, *p* < 0.001), with no significant main effects of ELS (F(1,40) = 0.25, *p* = 0.614) or drug (F(1,40) = 1.1, *p* = 0.3). An independent-samples *t*-test showed that nfκb1 expression was downregulated in the ELS-Vehicle group compared to both the NoELS-Vehicle (t(19) = −2.5, *p* = 0.022) and ELS-URB (t(23) = −3.781, *p* = 0.001) groups, suggesting that URB normalized the ELS-induced downregulation of nfκb1. Additionally, the NoELS-URB group showed downregulation of nfκb1 expression compared to the ELS-URB group (t(21) = 3.306, *p* = 0.003).

In females (Figure 2a, right), a two-way ANOVA revealed a significant main effect of ELS (F(1,34) = 4.73, *p* = 0.037), with no significant effects of drug (F(1,34) = 1.36, *p* = 0.25) or ELS × drug interaction (F(1,34) = 1.48, *p* = 0.232). ELS downregulated nfκb1 expression compared to the NoELS groups.

##### il1β

A three-way ANOVA revealed significant main effects of sex (F(1,64) = 27.66, *p* < 0.001), as well as significant interactions for ELS × drug (F(1,64) = 9.33, *p* = 0.003) and ELS × drug × sex (F(1,64) = 16.32, *p* < 0.001). No significant effects were observed for ELS (F(1,64) = 0.02, *p* = 0.876), drug (F(1,64) = 0.45, *p* = 0.501), ELS × sex interaction (F(1,64) = 0.09, *p* = 0.76), or sex × drug interaction (F(1,64) = 1.38, *p* = 0.244).

In males (Figure 2b, left), a two-way ANOVA revealed no significant effects of ELS (F(1,39) = 0.01, *p* = 0.92), drug (F(1,39) = 1.54, *p* = 0.222), or the ELS × drug interaction (F(1,39) = 0.43, *p* = 0.513).

In females (Figure 2b, right), a two-way ANOVA identified a significant ELS × drug interaction (F(1,25) = 44.24, *p* < 0.001), while no significant effects were found for ELS (F(1,25) = 0.18, *p* = 0.668) or drug (F(1,25) = 0.22, *p* = 0.644). Independent-samples *t*-tests indicated a notable upregulation of il1β in the ELS-Vehicle group compared to both the NoELS-Vehicle (t(13) = 5.207, *p* < 0.001) and ELS-URB (t(14) = 5.867, *p* < 0.001) groups, suggesting that URB treatment normalized the ELS-induced upregulation of il1β. Furthermore, the NoELS-URB group showed increased il1β expression compared to both the NoELS-Vehicle (t(11) = −3.757, *p* = 0.003) and ELS-URB (t(12) = −4.23, *p* = 0.001) groups, indicating a differential effect of URB in stressed versus non-stressed females.

##### il6

A three-way ANOVA indicated a significant effect of sex (F(1,64) = 102.25, *p* < 0.001). However, no significant effects were observed for ELS (F(1,64) = 0.473, *p* = 0.494), drug (F(1,64) = 0.60, *p* = 0.441), ELS × sex interaction (F(1,64) = 0.57, *p* = 0.451), sex × drug interaction (F(1,64) = 2.55, *p* = 0.115), ELS × drug interaction (F(1,64) = 2.32, *p* = 0.132), or ELS × drug × sex interaction (F(1,64) = 2.23, *p* = 0.14).

In males (Figure 2c, left), a two-way ANOVA revealed no significant effects for ELS (F(1,37) = 0.003, *p* = 0.959), drug (F(1,37) = 3.56, *p* = 0.554), or ELS × drug interaction (F(1,37) = 0.00, *p* = 0.983).

In females (Figure 2c, right), a two-way ANOVA revealed a significant ELS × drug interaction (F(1,27) = 4.71, *p* = 0.039), with no significant effects for ELS (F(1,27) = 1.08, *p* = 0.308) or drug (F(1,27) = 2.91, *p* = 0.099). An independent-samples *t*-test showed downregulation of il6 in the ELS-Vehicle group compared to both the NoELS-Vehicle (t(14) = −2.49, *p* = 0.026) and ELS-URB (t(17) = −3.943, *p* = 0.003) groups, suggesting that URB normalized the ELS-induced downregulation of il6.

##### tnfα

A three-way ANOVA revealed significant effects of sex (F(1,75) = 46.35, *p* < 0.001) and ELS (F(1,75) = 4.99, *p* = 0.028). However, no significant effects were found for drug (F(1,75) = 2.72, *p* = 0.103), ELS × sex interaction (F(1,75) = 0.61, *p* = 0.435), sex × drug interaction (F(1,75) = 0.09, *p* = 0.766), ELS × drug interaction (F(1,75) = 0.25, *p* = 0.618), or ELS × drug × sex interaction (F(1,75) = 3.47, *p* = 0.066).

In males (Figure 2d, left), a two-way ANOVA indicated no significant effects for ELS (F(1,44) = 1.09, *p* = 0.302), drug (F(1,44) = 0.95, *p* = 0.335), or ELS × drug interaction (F(1,44) = 0.96, *p* = 0.331).

In females (Figure 2d, right), a two-way ANOVA revealed a significant effect of ELS (F(1,31) = 4.62, *p* = 0.039), while no significant effects were observed for drug (F(1,31) = 1.92, *p* = 0.175) or ELS × drug interaction (F(1,31) = 2.83, *p* = 0.102).

For descriptive statistics and summary, see SI Tables S3 and S4, respectively. 

##### Correlations Between the Expression of Inflammatory Genes in the mPFC and Behavioral Responses in Adult Males and Females

Pearson correlations examined the association between behavioral responses and the expression of inflammatory genes (nfκb1, il1β, il6, and tnfα) in the mPFC of adult males and females, focusing on anxiogenic- and depressive-like phenotypes. 

In males (see Table S5), mPFC-il1β expression positively correlated with swimming behavior in the FST (r = 0.427, *p* = 0.03), indicating that lower mPFC-il1β levels are associated with reduced active coping. 

In females (see Table S6), mPFC-nfκb1 (immobility: r = 0.464, *p* = 0.013; swimming: r = −0.407, *p* = 0.032) and mPFC-tnfα (immobility: r = 0.382, *p* = 0.049; swimming: r = −0.434, *p* = 0.024) were positively correlated with immobility and negatively correlated with swimming in the FST, indicating that lower expression levels of these genes are associated with less learned helplessness behavior. In contrast, mPFC-il1β (r = 0.522, *p* = 0.015) was positively correlated with immobility, indicating that elevated expression is associated with increased learned helplessness. 

#### 3.1.3. Chronic Late Adolescence URB597 Treatment Ameliorates ELS-Induced Changes in Stress-Related Gene Expression in the mPFC

Studies have shown that rodents exposed to early life stressors, such as maternal separation, exhibit increased NF-κB activity in the PFC [40]. Given our finding that ELS reduced nfκb1 levels in both sexes, we further examined the effects of ELS and URB treatment on stress-related gene expression (nr3c1 and crf) in the mPFC, as the NF-κB pathway can be modulated by glucocorticoids [29]. For descriptive statistics, see SI Table S7.

##### nr3c1

A three-way ANOVA revealed a significant effect of sex (F(1,69) = 644.43, *p* < 0.001). In contrast, no significant effects were observed for ELS (F(1,69) = 0.301, *p* = 0.585), drug (F(1,69) = 0.15, *p* = 0.701), ELS × sex interaction (F(1,69) = 0.09, *p* = 0.76), sex × drug interaction (F(1,69) = 0.27, *p* = 0.6), ELS × drug interaction (F(1,69) = 0.906, *p* = 0.345), or ELS × drug × sex interaction (F(1,69) = 2.91, *p* = 0.092).

**Figure 3:**
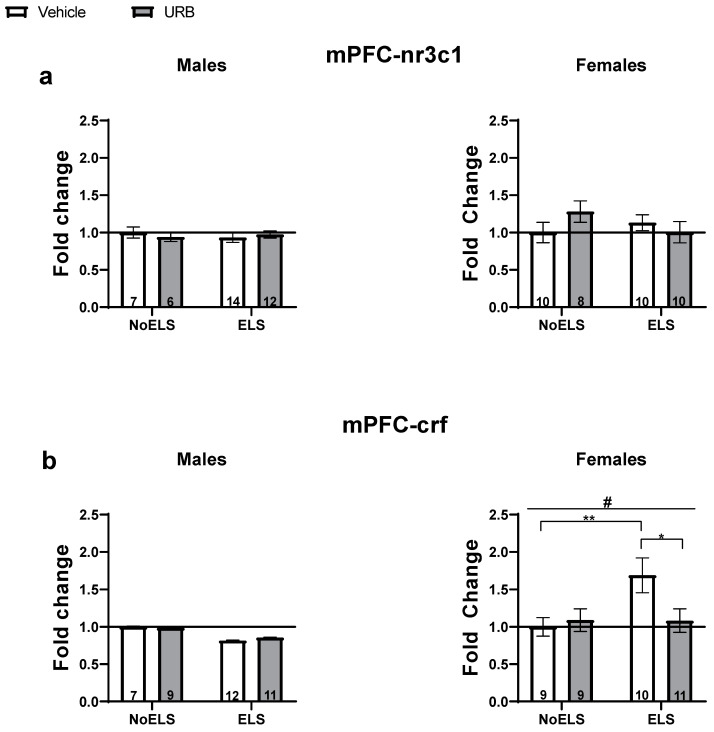
Chronic URB597 treatment during late adolescence ameliorates ELS-induced changes in stress-related gene expression in the mPFC. (**a**) No significant differences were found in nr3c1 expression in either males (**left**) or females (**right**). (**b**) In males (**left**), no significant differences were found in crf expression. In females (**right**), URB treatment normalized the ELS-induced crf upregulation. ELS: early life stress; mPFC: medial prefrontal cortex; URB: URB597. *, *p* < 0.05; **, *p* < 0.01 indicate statistically significant effects followed by post hoc comparisons; #, *p* < 0.05 indicate statistical significance in main effects.

In males (Figure 3a, left), a two-way ANOVA revealed no significant effects of ELS (F(1,35) = 0.05, *p* = 0.813), drug (F(1,35) = 0.02, *p* = 0.891), or ELS × drug interaction (F(1,35) = 0.55, *p* = 0.463).

In females (Figure 3a, right), a two-way ANOVA also showed no significant effects for ELS (F(1,34) = 0.25, *p* = 0.619), drug (F(1,34) = 0.28, *p* = 0.595), or ELS × drug interaction (F(1,34) = 2.43, *p* = 0.128).

##### crf

A three-way ANOVA with a log link function was conducted due to the non-normal distribution of the variable. Significant effects were observed for sex (X^2^(1) = 259.63, *p* < 0.001) and the ELS × sex interaction (X^2^(1) = 4.33, *p* = 0.037). No significant effects were found for ELS (X^2^(1) = 0.002, *p* = 0.96), drug (X^2^(1) = 0.43, *p* = 0.51), sex × drug interaction (X^2^(1) = 0.704, *p* = 0.402), ELS × drug interaction (X^2^(1) = 0.82, *p* = 0.363), or ELS × drug × sex interaction (X^2^(1) = 1.59, *p* = 0.207).

In males (Figure 3b, left), a two-way ANOVA using a log link function did not reveal any significant effects for ELS (X^2^(1) = 1.38, *p* = 0.24), drug (X^2^(1) = 0.01, *p* = 0.916), or ELS × drug interaction (X^2^(1) = 0.04, *p* = 0.841).

In females (Figure 3b, right), a two-way ANOVA with a log link function identified significant effects for ELS (X^2^(1) = 3.95, *p* = 0.047) and the ELS × drug interaction (X^2^(1) = 4.15, *p* = 0.042), with no significant effect for drug (X^2^(1) = 1.97, *p* = 0.16). ELS was found to upregulate crf expression compared to the NoELS groups. An independent-samples *t*-test indicated a significant upregulation of crf in the ELS-Vehicle group compared to both the NoELS-Vehicle (t(35) = −2.764, *p* = 0.009) and ELS-URB (t(35) = −2.455, *p* = 0.019) groups, suggesting that URB normalized the ELS-induced upregulation of crf.

##### Correlations Between the Expression of Stress-Related Genes in the mPFC and Behavioral Responses in Adult Males and Females

Pearson correlations were used to examine the association between behavioral responses and the expression of stress-related genes (nr3c1 and crf) in the mPFC of adult males and females, focusing on anxiogenic- and depressive-like phenotypes. 

No significant correlations were found in males (see Table S8). 

In females (see Table S9), mPFC-nr3c1 positively correlated with immobility in the FST, indicating that higher levels of mPFC-nr3c1 are associated with increased learned helplessness. 

#### 3.1.4. Chronic Late Adolescence URB597 Treatment Ameliorates ELS-Induced Changes in Inflammatory Genes’ Expression in the Hippocampal CA1

##### nfκb1

A three-way ANOVA revealed significant effects of sex (F(1,75) = 63.04, *p* < 0.001) and the ELS × drug × sex interaction (F(1,75) = 4.31, *p* = 0.041). However, no significant effects were observed for ELS (F(1,75) = 0.15, *p* = 0.695), drug (F(1,75) = 1.14, *p* = 0.288), ELS × sex interaction (F(1,75) = 2.09, *p* = 0.153), sex × drug interaction (F(1,75) = 2.63, *p* = 0.109), or ELS × drug interaction (F(1,75) = 0.32, *p* = 0.571).

**Figure 4:**
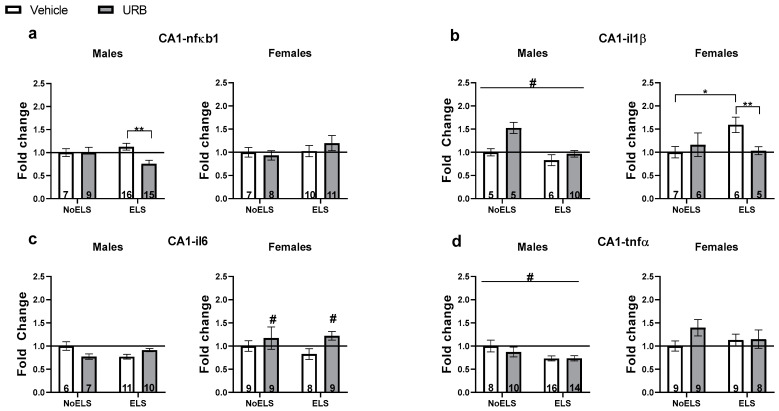
Chronic late adolescence URB597 treatment ameliorates ELS-induced changes in inflammatory gene expression in the hippocampal CA1. (**a**) In ELS males (**left**), URB treatment downregulated nfκb1 expression. In females (**right**), no significant differences were found in nfκb1 expression. (**b**) In males (**left**), ELS downregulated il1β expression. In females (**right**), URB treatment normalized ELS-induced il1β upregulation. (**c**) In males (**left**), no significant differences were found in il6 expression. In females (**right**), URB treatment upregulated il6 expression compared to the vehicle groups. (**d**) In males (**left**), ELS downregulated tnfα expression. In females (**right**), no significant differences were found in tnfα expression. ELS: early life stress; URB: URB597. *, *p* < 0.05; **, *p* < 0.01 indicate statistically significant effects followed by post hoc comparisons; #, *p* < 0.05 indicate statistical significance in main effects.

In males (Figure 4a, left), a two-way ANOVA revealed a significant effect of drug (F(1,43) = 4.403, *p* = 0.042) and the ELS × drug interaction (F(1,43) = 4.25, *p* = 0.045), while no significant effect of ELS was found (F(1,43) = 0.67, *p* = 0.417). An independent-samples *t*-test indicated a significant downregulation of nfκb1 in the ELS-URB group compared to the ELS-Vehicle group (t(29) = 3.541, *p* = 0.001).

In females (Figure 4a, right), a two-way ANOVA showed no significant effects for ELS (F(1,32) = 1.37, *p* = 0.25), drug (F(1,32) = 0.12, *p* = 0.727), or the ELS × drug interaction (F(1,32) = 0.92, *p* = 0.343).

##### il1β

A three-way ANOVA revealed significant effects for sex (F(1,43) = 52.87, *p* < 0.001), the ELS × sex interaction (F(1,43) = 5.79, *p* = 0.02), the sex × drug interaction (F(1,43) = 4.64, *p* = 0.037), and the ELS × drug interaction (F(1,43) = 4.22, *p* = 0.046). In contrast, no significant effects were found for ELS (F(1,43) = 0.47, *p* = 0.494), drug (F(1,43) = 0.59, *p* = 0.446), or the ELS × drug × sex interaction (F(1,43) = 0.66, *p* = 0.42).

In males (Figure 4b, left), a two-way ANOVA indicated a significant effect of ELS (F(1,23) = 4.46, *p* = 0.046), while no significant effects were observed for drug (F(1,23) = 3.97, *p* = 0.058) or the ELS × drug interaction (F(1,23) = 0.71, *p* = 0.406). Specifically, ELS was associated with a downregulation of il1β expression compared to the NoELS group.

In females (Figure 4b, right), a two-way ANOVA showed a significant effect of the ELS × drug interaction (F(1,20) = 4.53, *p* = 0.046), with no significant effects for ELS (F(1,20) = 1.62, *p* = 0.217) or drug (F(1,20) = 1.05, *p* = 0.316). An independent-samples *t*-test revealed that il1β expression was significantly upregulated in the ELS-Vehicle group compared to both the NoELS-Vehicle (t(11) = 2.876, *p* = 0.015) and ELS-URB (t(9) = 3.4, *p* = 0.008) groups, suggesting that URB treatment effectively normalized the ELS-induced upregulation of il1β.

##### il6

A three-way ANOVA identified significant effects of sex (F(1,60) = 45.97, *p* < 0.001) and the ELS × drug interaction (F(1,60) = 4.45, *p* = 0.039). However, no significant effects were found for ELS (F(1,60) = 0.302, *p* = 0.585), drug (F(1,60) = 2.26, *p* = 0.137), the ELS × sex interaction (F(1,60) = 0.102, *p* = 0.75), the sex × drug interaction (F(1,60) = 2.91, *p* = 0.093), or the ELS × drug × sex interaction (F(1,60) = 0.56, *p* = 0.457).

In males (Figure 4c, left), a two-way ANOVA revealed a significant ELS × drug interaction (F(1,30) = 5.208, *p* = 0.03), with no significant effects for ELS (F(1,30) = 0.03, *p* = 0.856) or drug (F(1,30) = 0.02, *p* = 0.874). An independent-samples *t*-test did not identify any significant differences.

In females (Figure 4c, right), a two-way ANOVA indicated a significant effect of drug (F(1,30) = 4.29, *p* = 0.047), while no significant effects were observed for ELS (F(1,30) = 0.31, *p* = 0.579) or the ELS × drug interaction (F(1,30) = 0.77, *p* = 0.386). URB treatment was found to upregulate il6 expression compared to the vehicle treatment groups.

##### tnfα

A three-way ANOVA revealed a significant main effect of sex (F(1,75) = 69.48, *p* < 0.001), while no significant effects were observed for ELS (F(1,75) = 3.37, *p* = 0.07), drug (F(1,75) = 0.507, *p* = 0.479), ELS × sex interaction (F(1,75) = 1.83, *p* = 0.179), sex × drug interaction (F(1,75) = 2.52, *p* = 0.116), ELS × drug interaction (F(1,75) = 0.34, *p* = 0.558), or ELS × drug × sex interaction (F(1,75) = 2.31, *p* = 0.133).

In males (Figure 4d, left), a two-way ANOVA showed a significant main effect of ELS (F(1,44) = 6.39, *p* = 0.015), with no significant effects of drug (F(1,44) = 0.48, *p* = 0.491) or the ELS × drug interaction (F(1,44) = 0.54, *p* = 0.464). ELS was associated with the downregulation of tnfα expression compared to the NoELS groups.

In females (Figure 4d, right), a two-way ANOVA indicated no significant main effects of ELS (F(1,31) = 0.09, *p* = 0.765), drug (F(1,31) = 2.08, *p* = 0.159), or the ELS × drug interaction (F(1,31) = 1.75, *p* = 0.196).

For descriptive statistics and summary, see SI Table S10 and S11, respectively. 

##### Correlations Between Inflammatory Gene Expression in the Hippocampal CA1 and Behavior in Adult Males and Females

Pearson bivariate correlation tests were performed. 

In males (see Table S12), lower CA1-tnfα levels were correlated with increased immobility, suggesting learned helplessness (r = −0.395, *p* = 0.038). 

In females (see Table S13), CA1-nfκb1 positively correlated with distance traveled in the OFT, indicating that higher CA1-nfκb1 levels were associated with increased locomotion (r = 0.491, *p* = 0.009). Also, negative correlations were found between higher CA1-nfκb1 expression levels and impaired short-term memory in the SR test (r = −0.38, *p* = 0.05), and higher CA1-il1β levels were associated with decreased sociability in the SP test (r = −0.484, *p* = 0.026).

### 3.2. Experiment 2: ELS-Induced Alterations in Inflammatory and Stress-Related Genes in the mPFC and Hippocampal CA1 of Late Adolescent Rats, Prior to URB Treatment

**Figure 5:**
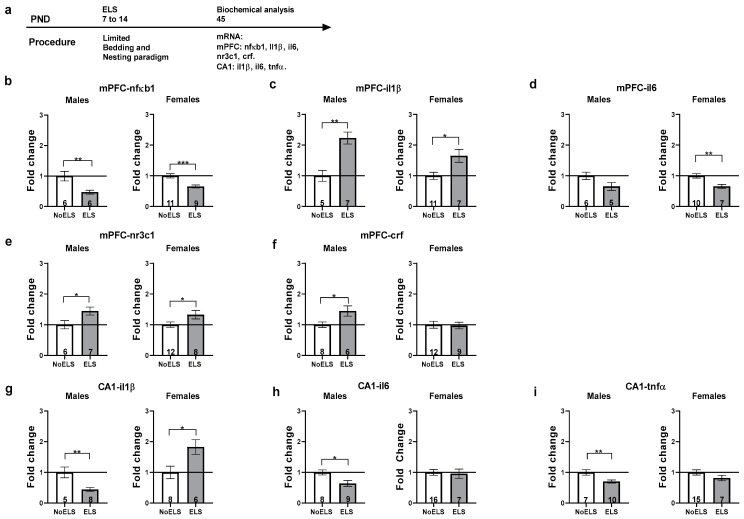
ELS-induced protracted changes in inflammatory and stress-related genes in the mPFC and hippocampal CA1. (**a**) Male and female rats were exposed to early life stress (ELS; P7-14). On P45, rats were sacrificed, and brains were harvested for molecular analysis (n = 5–16 for all groups). (**b**) ELS downregulated mPFC-nfκb1 expression in both males (**left**) and females (**right**). (**c**) ELS upregulated mPFC-il1β expression in both males (**left**) and females (**right**). (**d**) No significant differences were found in mPFC-il6 expression in males (**left**). In females (**right**), ELS downregulated mPFC-il6 expression. (**e**) ELS upregulated mPFC-nr3c1 expression in both males (**left**) and females (**right**). (**f**) ELS upregulated mPFC-crf expression in males (**left**). No significant differences were found in mPFC-crf expression in females (**right**). (**g**) ELS downregulated CA1-il1β expression in males (**left**) but upregulated CA1-il1β expression in females (**right**). (**h**) ELS downregulated CA1-il6 expression in males (**left**). No significant differences were found in CA1-il6 expression in females (**right**). (**i**) ELS downregulated CA1-tnfα expression in males (**left**). No significant differences were found in CA1-tnfα expression in females (**right**). ELS: early life stress; mPFC: medial prefrontal cortex; mRNA: messenger RNA; *p*: post-natal day. *, *p* < 0.05; **, *p* < 0.01; ***, *p* < 0.001 indicate statistically significant effects followed by post hoc comparisons.

Given the observed ELS-induced alterations in inflammatory and stress-related genes in the mPFC and CA1 of adult rats, we aimed to determine whether these changes were already present at P45, prior to the initiation of URB treatment. To this end, we focused on genes that were altered in the ELS-Vehicle groups and subsequently normalized by URB in adulthood within both brain regions. This approach ensured that URB’s effects were specific to genes affected by stress exposure and that URB could reverse persistent inflammatory marker alterations. In parallel with the first experiment, stress-related genes were exclusively examined in the mPFC. At P45, male and female rats exposed to ELS were sacrificed, and their brains were extracted for biochemical analysis (see Figure 5a for experimental design, n = 5–16 per sex in each group).

#### 3.2.1. mPFC-nfκb1

Independent-samples *t*-tests revealed that ELS downregulated nfκb1 expression compared to the NoELS group in both sexes [males (Figure 5b, left): (t_(10)_ = −4.001, *p* = 0.003); females (Figure 5b, right): (t_(18)_ = −4.684, *p* < 0.001)].

#### 3.2.2. mPFC-il1β

ELS upregulated il1β expression compared to the NoELS group in both sexes [males (Figure 5c, left): (t_(10)_ = 4.206, *p* = 0.002); females (Figure 5c, right): (t_(16)_ = 2.922, *p* = 0.01)].

#### 3.2.3. mPFC-il6

No significant differences were observed in males (Figure 5d, left). However, in females (Figure 5d, right), ELS downregulated il6 expression compared to the NoELS group (t_(15)_ = −3.313, *p* = 0.007). 

#### 3.2.4. mPFC-nr3c1

ELS upregulated nr3c1 expression compared to the NoELS group in both sexes [males (Figure 5e, left): (t_(11)_ = 2.333, *p* = 0.04); females (Figure 5e, right): (t_(17)_ = 2.507, *p* = 0.023)].

#### 3.2.5. mPFC-crf

ELS upregulated crf expression compared to the NoELS group in males (t_(12)_ = 2.72, *p* = 0.019). No significant difference was found between the groups in females (Figure 5f, right).

#### 3.2.6. CA1-il1β

ELS downregulated il1β expression compared to the NoELS group in males (Figure 5g, left) (t_(11)_ = −3.725, *p* = 0.003) but upregulated il1β expression in females (Figure 5g, right) compared to the NoELS group (t_(12)_ = 2.779, *p* = 0.017). 

#### 3.2.7. CA1-il6

ELS downregulated il6 expression compared to the NoELS group in males (Figure 5h, left) (t_(15)_ = −2.734, *p* = 0.015). No significant difference was found between the groups in females (Figure 5h, right).

#### 3.2.8. CA1-tnfα

ELS downregulated tnfα expression compared to the NoELS group in males (Figure 5i, left) (t_(15)_ = −3.394, *p* = 0.004). No significant difference was found between the groups in females (Figure 5i, right).

## 4. Discussion

Our study reveals that ELS induces a depressive-like phenotype in both sexes, which is effectively reversed by the FAAH inhibitor. Additionally, ELS causes enduring changes in neuroinflammatory markers in the mPFC and hippocampal CA1 region, with these effects being sex dependent. Notably, URB597 normalized ELS-induced changes in nfκb1 expression in males and il1β and il6 expression in females within the mPFC, as well as il1β expression in females within the CA1. 

### 4.1. Impact of ELS and URB597 Treatment on Behavioral Outcome in Adult Male and Female Rats

Our findings indicate that exposure to ELS led to a depressive-like phenotype in adult rats of both sexes. In males, ELS exposure led to reduced locomotion, decreased swimming behavior, and increased immobility. In females, ELS decreased swimming behavior and increased immobility. Treatment with URB597 during late adolescence reversed these effects in both sexes. These results align with previous studies from our lab, which validate the LBN paradigm for assessing the association between ELS and depressive-like phenotype in adulthood and demonstrate that the FAAH inhibitor URB can counteract depressive-like symptoms induced by ELS [27,33,34,38].

URB treatment decreased locomotion in the OFT and led to increased immobility in the FST in non-stressed female rats. These observations align with our previous study [27] and suggest that URB may differentially affect behavior in non-stressed females. This is consistent with research showing that cannabinoid agonists more effectively reduce locomotor activity in female compared to male rats [41,42]. The observed differences may be attributed to variation in endocannabinoid signaling and CB1 receptor expression, with females exhibiting increased G protein activation in the PFC, which could explain these differing responses [43,44]. 

### 4.2. Impact of ELS and URB597 on Inflammatory and Stress-Related Gene Expression in the mPFC 

ELS resulted in a persistent downregulation of nfκb1 expression in both sexes, starting at P45. NF-κB, a key transcriptional regulator of inflammatory genes [45], plays a significant role in stress-related neurogenesis and depressive behavior [46] and has been associated with ELS [40]. However, previous research showed that maternal separation in mice is linked to elevated mPFC-nfκb1 levels and impaired working memory [40]. This discrepancy may be due to differences in experimental paradigms, suggesting that various stress models can distinctly affect gene expression. 

A possible explanation for the downregulation of mPFC-nfκb1 may involve the interaction between NF-κB and stress-related pathways, such as those mediated by glucocorticoid receptors (GRs). This interaction could disrupt NF-κB signaling, reflecting the impact of chronic stress on neuroinflammatory processes. GRs, known for their anti-inflammatory and immunomodulatory roles, modulate transcription factors like NF-κB [29,47]. Our study found that ELS upregulated mPFC-nr3c1 expression in both sexes during late adolescence (P45), suggesting that elevated GR levels following ELS may have contributed to the persistent downregulation of mPFC-nfκb1 expression. Chronic exposure to stress, such as ELS, can result in long-term adaptations in the stress response system. Hence, over time, the persistent activation of GRs might lead to sustained suppression of NF-κB activity, which could manifest as a persistent downregulation of mPFC-nfκb1. This adaptation might be a mechanism by which the brain attempts to counteract the chronic inflammatory effects of stress. Also, ELS upregulated mPFC-crf expression in adult females and in males at P45. However, these changes in males did not persist into adulthood, suggesting a transient activation of the stress response system during a critical period of development. 

Notably, URB treatment selectively normalized mPFC-nfκb1 expression in males, suggesting a distinct sex-dependent mechanism underlying ELS-induced neuroinflammatory changes. This finding aligns with previous studies showing URB’s ability to normalize nfκb1 levels in other models where they are upregulated. For example, Su et al. (2017) [22] demonstrated that URB alleviated neuroinflammation induced by chronic cerebral hypoperfusion (CCH) by inhibiting NF-κB signaling in the mPFC and hippocampus. Similarly, Rivera et al. (2018) [24] reported that URB reduced nfκb1 levels in the hippocampus of rats exposed to ethanol. These studies suggest that URB can effectively normalize both upregulated and downregulated mPFC-nfκb1 levels in male rats.

In contrast to males, ELS affected females by downregulating il6 and tnfα expression while upregulating il1β and crf expression. The effects on tnfα and crf emerged only in adulthood. The persistent reduction in il6 and increased il1β in the mPFC levels from P45 suggests a potential disruption or alteration in the inflammatory response that could be indicative of a long-lasting impact of ELS on neuroinflammatory pathways. This finding contrasts with studies using maternal separation as a model of ELS, which report an upregulation of il6 and tnfα in the brain, particularly in the mPFC and hippocampus [17,18]. This discrepancy may highlight differences in how various models of ELS influence inflammatory processes, suggesting that the effects of ELS on inflammatory markers like il6 might vary depending on the specific stress model and the timing of its impact. Interestingly, Burke et al. (2013) [48] also found that a single day of maternal separation decreased mPFC-tnfα levels only in females. Also, the alterations observed in females in nfκb1, il1β, and tnfα were significantly associated with learned helplessness behavior. The observed increase in il1β in both sexes and in crf levels in males at P45 aligns with other studies reporting elevated il1β and CRF levels following ELS. For example, Wang et al. (2020) [49] found increased il1β levels in the mPFC of adult rats exposed to 19 days of maternal separation. Similarly, Burke et al. (2013) [48] observed elevated mPFC-il1β levels after just one day of maternal deprivation. Additionally, a review by Pryce et al. (2002) [50] also noted that adult rats exposed to early-life maternal separation generally exhibit increased CRF levels. Interestingly, URB treatment normalized the decrease in mPFC-il6 and the increases in mPFC-il1β and mPFC-crf levels in stressed female rats. The normalization of mPFC-il6 is consistent with previous studies demonstrating URB’s ability to regulate il6 levels, whether elevated or decreased [51]. For example, URB was effective in reducing elevated il6 in the hippocampus of female rats exposed to chronic unpredictable stress. The attenuation of mPFC-il1β and mPFC-crf increases also aligns with previous findings. URB administration (1 mg/kg, i.p.) thirty minutes before lipopolysaccharide (LPS) injection reduced the LPS-induced rise in il1β expression in the hypothalamus [52]. Additionally, CB1 receptor-dependent mechanisms in the mPFC are proposed to mediate the cessation of HPA axis activation following stress [53]. CB1 receptor mRNA has been co-localized with CRF mRNA in both the paraventricular nucleus of the hypothalamus and other extrahypothalamic areas of the brain’s fear circuit, such as the mPFC and amygdala [54]. 

Additionally, URB treatment increased mPFC-il1β levels in non-stressed female rats. Elevated mPFC-il1β levels are associated with increased learned helplessness, as evidenced by the greater immobility observed in the FST among non-stressed rats treated with URB. This suggests that URB can influence inflammatory processes even in the absence of stress. URB’s effects in non-stressed female rats may be mediated through the enhancement of mPFC-il1β levels. The fact that URB increases mPFC-il1β levels in non-stressed rats, but may have a different effect in stressed rats, highlights the complexity of URB’s impact. It suggests that the effects of URB on mPFC-il1β and associated behaviors can vary depending on the stress status of the individual.

### 4.3. Impact of ELS and URB597 on Inflammatory and Stress-Related Gene Expression in the CA1 Region 

ELS resulted in persistent downregulation of CA1-il1β and CA1-tnfα in males, with these changes observed already at P45. The reduction in CA1-tnfα expression was associated with increased learned helplessness. Although these findings contrast with the existing literature demonstrating upregulation after maternal separation [48,49], the persistence of these alterations suggests a long-term adaptation or modulation of inflammatory processes in the hippocampus, which could have significant implications for hippocampal function and stress-related outcomes. 

In females, ELS resulted in a persistent upregulation of CA1-il1β expression starting from P45. Importantly, this upregulation was reversed with URB treatment, further supporting the therapeutic potential of URB in mitigating the neuroinflammatory effects of ELS. Our finding is consistent with studies showing that ELS increases IL1β expression in the hippocampus [17,49].

### 4.4. Sex Differences and ECS

The sex differences observed in this study may be attributed to a combination of biological and hormonal factors that differentially influence how males and females respond to ELS, URB597 treatment, and neuroinflammatory processes. Estrogen and Progesterone hormones can modulate endocannabinoid signaling. Estrogen, in particular, has been shown to influence endocannabinoid levels and receptor expression, contributing to sex differences in how cannabinoids affect the brain [55,56]. Estrogen affects endocannabinoid signaling by influencing CB1 receptors expression in the central nervous system and increasing AEA levels through reduced FAAH transcription in both sexes [55]. However, because estrogens have varying effects on endocannabinoid signaling across different tissues, it is likely that multiple interaction pathways are involved and can differentially affect behavior and response to cannabinoid treatment. 

## 5. Conclusions

Our study demonstrates that ELS induces a depressive-like phenotype in both sexes, which can be effectively reversed by the FAAH inhibitor URB597. ELS leads to lasting changes in neuroinflammatory markers in the mPFC and hippocampal CA1 region, with these effects varying by sex. Importantly, URB597 alleviates some of these neuroinflammatory changes in the mPFC and CA1, suggesting that its antidepressant-like effects are mediated through the modulation of inflammatory cytokines, and these effects differ between males and females. 

Overall, our findings underscore the potential of URB597 as a therapeutic strategy for addressing ELS-induced depressive-like behavior through the modulation of neuroinflammatory pathways. The observed sex differences highlight the importance of considering sex as a biological variable when evaluating the efficacy of treatments for depression. 

## Data Availability

Data is contained within the article or Supplementary Information.

## Supplementary Materials

The following supporting information can be downloaded at: https://www.mdpi.com/article/10.3390/cells13221881/s1, Figure S1: Brain areas for molecular analysis; Figure S2: Protracted effects of ELS on total exploration time in social tests for adult males and females; Figure S3: Long-term effects of ELS on climbing in the forced swim test (FST); Table S1: Primers for mRNAs used for real-time PCR; Table S2: Descriptive statistics of the behavioral tests; Table S3: Descriptive statistics of inflammatory gene expression in the mPFC; Table S4: Chronic late adolescence URB597 treatment ameliorates ELS-induced changes in inflammatory gene expression in the mPFC; Table S5: Correlations between the expression of inflammatory genes in the mPFC and behavioral responses in adult males; Table S6: Correlations between the expression of inflammatory genes in the mPFC and behavioral response in adult females; Table S7: Descriptive statistics of stress-related gene expression in the mPFC; Table S8: Correlations between the expression of stress-related genes in the mPFC and behavioral responses in adult males; Table S9: Correlations between the expression of stress-related genes in the mPFC and behavioral response in adult females; Table S10: Descriptive statistics of inflammatory gene expression in the hippocampal CA1; Table S11: Chronic late adolescence URB597 treatment ameliorates ELS-induced changes in inflammatory gene expression in the hippocampal CA1; Table S12: Correlations between the expression of inflammatory genes in the hippocampal CA1 and behavioral response in adult males; Table S13: Correlations between the expression of inflammatory genes in hippocampal CA1 and behavioral responses in adult females.
