# Supplementary material for: FAAH Inhibition Reverses Depressive-like Behavior and Sex-Specific Neuroinflammatory Alterations Induced by Early Life Stress
Source: Cells. 2024 Nov 14;13(22):1881. doi: 10.3390/cells13221881 (PMC11593135; doi:10.3390/cells13221881)
Supplement: Supplementary file 1 [file cells-13-01881-s001.zip › cells-3263978-supplementary.pdf]

Supplementary information

Material and methods

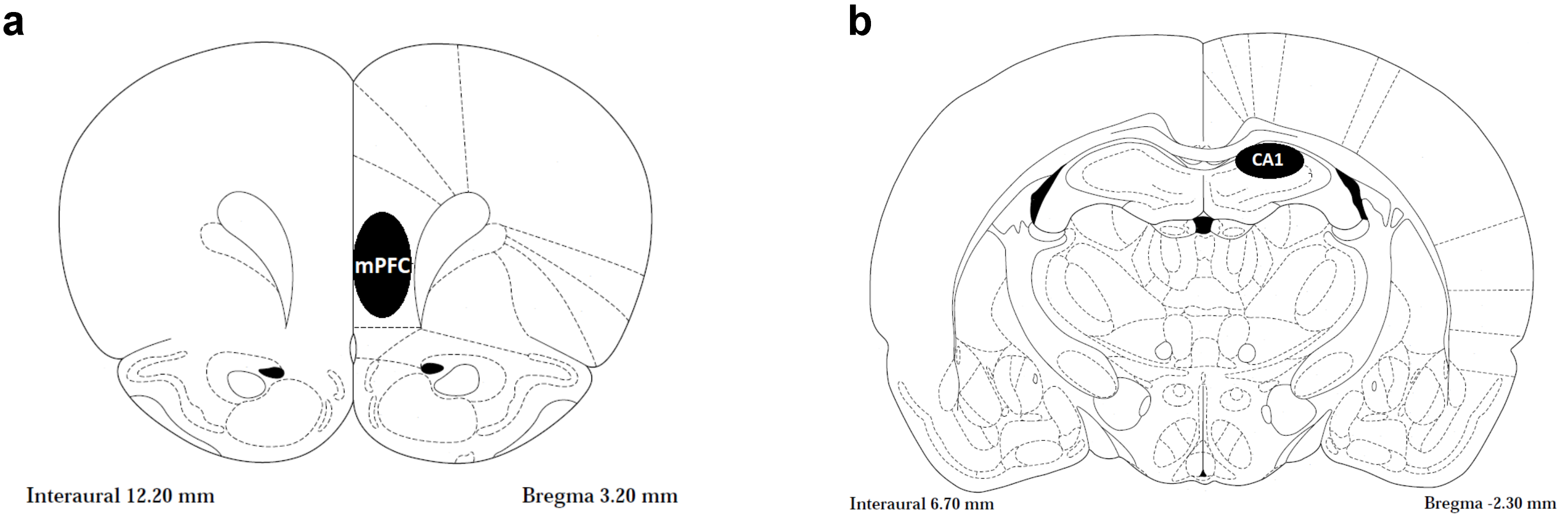

Figure S1. Brain areas for molecular analysis. Atlas illustration in coronal view; samples were obtained by punches (1 mm diameter). The numbers indicate the distance from Bregma: (a) mPFC at position 3.20 mm anterior to bregma. (b) CA1 region at position -2.30 mm posterior to bregma. mPFC: medial prefrontal cortex.

Table S1. Primers for mRNAs used for real-time PCR.

Name	Description	Gene Bank ID (NM)	Protein Name	Primer Sequence
hprt	Housekeeping gene; used as a reference gene	NM_012583.2	HPRT	F: 5′GAGCACTTCAGGGATTTGAATCA3′<br>R: 5′GTAGATTCAACTTGCCGCTGCTGTCT3′
nfκb1	Nuclear factor kappa B, subunit 1	NM_ 01415012.1	NFκB	F: 5′GAGCTCCCCATCTTCAAC3′<br>R: 5′ATTTGCCCAGTTCCGAAAGGATC3′
Il1β	Interleukin 1 beta	NM_031512.2	IL-1beta	F: 5′GCTGTGGCAGCTACCTATGTCTT3′<br>R: 5′GTCACAGAGGACGGGCTCTTC3′
Il6	Interleukin 6	NM_012589.2	IL-6	F: 5′CTTCCAAACTGGATATAACCAGG3′<br>R: 5′CTTCACAAACTCCAGGTAGAAAC3′
tnf*α*	Tumor necrosis factor alpha	NM_012675.3	TNF-alpha	F: 5′CCAGACCCTCACACTCAGATC3′<br>R: 5′CTCCGCTTGGTGGTTTGCTA3′
crf	Corticotrophin releasing factor	NM_031019.1	CRF	F: 5′CTGCCAAGGGAGGAGAAGAGA3′<br>R: 5′GAGACGGATCCCCTGCTCA3′
nr3c1	Nuclear receptor subfamily 3, group C, member 1	NM_012576.2	GR	F: 5′ACTGGAATAGGTGCCAAGGCT3′<br>R: 5′TGGTCTCATTCCAGGGCTTG3′

F: forward primer; GR: glucocorticoid receptor; R: reverse primer.

Results

For total exploration time in the social preference (SP) test (Figure S2a), a three-way ANOVA revealed significant effects of sex ($F_{(1,64)} = 27.157$, $p < 0.001$) and ELS ($F_{(1,64)} = 9.157$, $p = 0.004$), with no significant effects of drug ($F_{(1,64)} = 3.312$, $p = 0.073$), ELS × sex ($F_{(1,64)} = 0.66$, $p = 0.419$), sex × drug ($F_{(1,64)} = 0.025$, $p = 0.87$), ELS × drug ($F_{(1,64)} = 0.128$, $p = 0.72$) or ELS × drug × sex ($F_{(1,64)} = 0.918$, $p = 0.34$) interactions.

In males (Figure S2a, left), a two-way ANOVA revealed a significant effect of ELS ($F_{(1,32)} = 7.75$, $p = 0.009$), with no significant effects of drug ($F_{(1,32)} = 2.057$, $p = 0.161$) or ELS × drug interaction ($F_{(1,32)} = 0.19$, $p = 0.667$). NoELS males exhibited greater exploration time in the social preference test compared to those in the ELS groups.

In females (Figure S2a, right), a two-way ANOVA did not reveal any significant effects of ELS ($F_{(1,32)} = 2.334$, $p = 0.136$), drug ($F_{(1,32)} = 1.31$, $p = 0.26$) or ELS × drug interaction ($F_{(1,32)} = 0.826$, $p = 0.37$).

For total exploration time in the social recognition (SR) test (Figure S2b), a three-way ANOVA did not reveal any significant effects of sex ($F_{(1,54)} = 0.23$, $p = 0.634$), ELS ($F_{(1,54)} = 0.05$, $p = 0.816$), drug ($F_{(1,54)} = 0.05$, $p = 0.823$), ELS × sex ($F_{(1,54)} = 0.262$, $p = 0.611$), sex × drug ($F_{(1,54)} = 0.243$, $p = 0.624$), ELS × drug ($F_{(1,54)} = 0.164$, $p = 0.687$) or ELS × drug × sex ($F_{(1,54)} = 0.319$, $p = 0.08$) interactions.

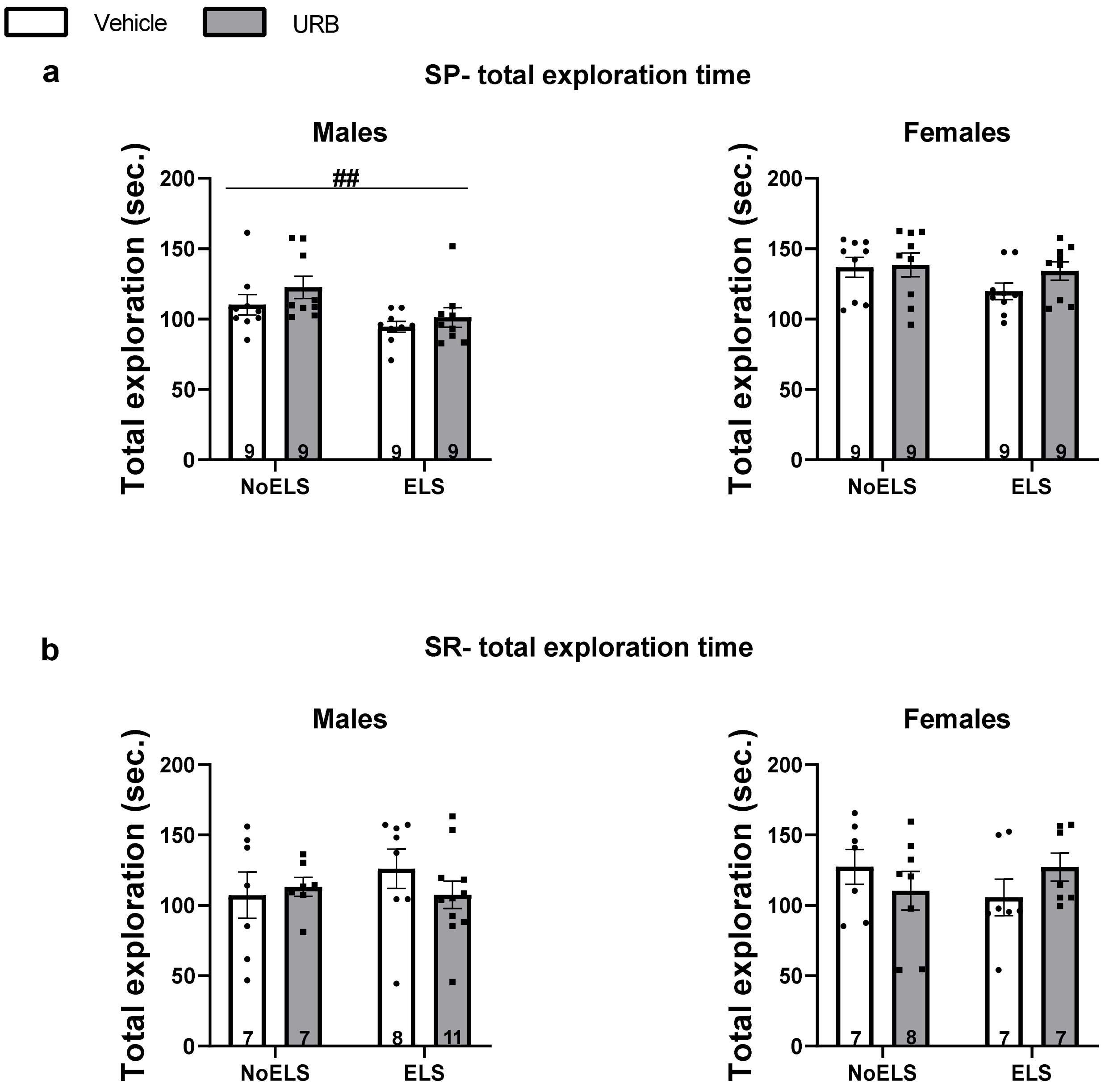

Figure S2. Protracted effects of ELS on total exploration time in social tests for adult males and females. (a) In the Social Preference (SP) test, ELS decreased total exploration time in males (left), compared to the NoELS groups, while no significant differences were observed in females (right). (b) In the Social Recognition (SR) test, no significant differences were observed in either sex. ELS: early life stress; URB: URB597. ##, $p < 0.01$ indicates statistical significance in main effects.

For climbing in the forced swim test (FST) (Figure S3), a three-way ANOVA revealed significant effect of sex × drug ($F_{(1,53)} = 5.926$, $p = 0.018$), with no significant effects of sex ($F_{(1,53)} = 3.814$, $p = 0.056$), ELS ($F_{(1,53)} = 1.227$, $p = 0.27$), drug ($F_{(1,53)} = 0.192$, $p = 0.66$), ELS × sex ($F_{(1,53)} = 0.839$, $p = 0.36$), ELS × drug ($F_{(1,53)} = 0.844$, $p = 0.72$) or ELS × drug × sex ($F_{(1,53)} = 0.005$, $p = 0.94$) interactions.

In males (Figure S3, left), a two-way ANOVA did not reveal any significant effects of ELS ($F_{(1,27)} = 0.015$, $p = 0.9$), drug ($F_{(1,27)} = 3.475$, $p = 0.07$) or ELS × drug interaction ($F_{(1,27)} = 0.415$, $p = 0.525$).

In females (Figure S3, right), a two-way ANOVA did not reveal any significant effects of ELS ($F_{(1,26)} = 2.566$, $p = 0.12$), drug ($F_{(1,26)} = 2.497$, $p = 0.12$) or ELS × drug interaction ($F_{(1,26)} = 0.447$, $p = 0.509$).

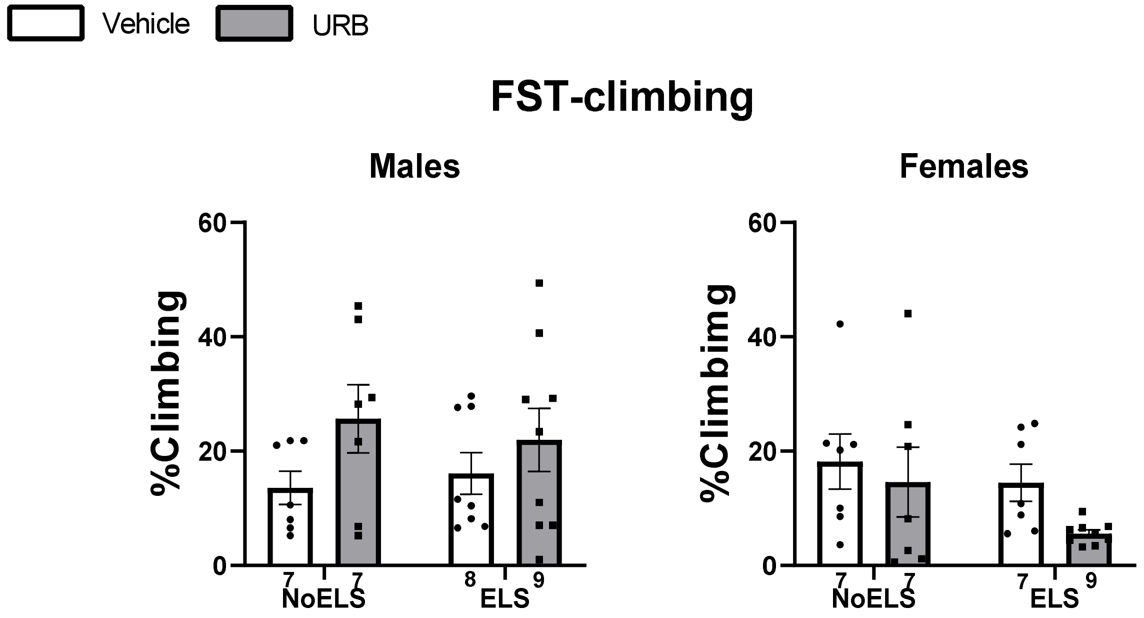

Figure S3. Long-term effects of ELS on climbing behavior in the Forced Swim Test (FST). No significant differences were observed in either sex. ELS: early life stress; URB: URB597.

Table S2. Descriptive statistics for the behavioral tests.

Behavioral test		NoELS-Vehicle	NoELS-URB	ELS-Vehicle	ELS-URB
Open Field Test (cm)	M: mean	6920.43	6087.7	5239.18	6248.65
	SEM	322.01	441.93	110.3	283.72
	F: mean	7968.82	6914.28	6993.1	7534.45
	SEM	205.87	257.09	369.06	286.95
Social Preference Test (DI)	M: mean	0.788	0.806	0.845	0.867
	SEM	0.01	0.01	0.01	0.01
	F: mean	0.852	0.763	0.741	0.858
	SEM	0.01	0.04	0.02	0.02
Social Recognition Test (DI)	M: mean	0.581	0.687	0.481	0.761
	SEM	0.05	0.04	0.05	0.01
	F: mean	0.666	0.613	0.612	0.692
	SEM	0.04	0.03	0.05	0.03
Forced Swim Test-Swimming (%)	M: mean	49.4	33.8	18.65	35.3
	SEM	3.5	7.6	3.03	4.9
	F: mean	38.2	27.7	35.4	57.4
	SEM	4	5.7	4.1	2.7
Forced Swim Test-Immobility (%)	M: mean	37	40.5	65.2	42.7
	SEM	2.8	4.9	0.9	2.2
	F: mean	43.5	57.7	50.08	37
	SEM	3.6	2.6	2.6	2.5

DI: discriminative index; ELS: early life stress; F: females; M: males; SEM: standard error of the mean; URB: URB597.

Table S3. Descriptive statistics of inflammatory gene expression in the mPFC.

Gene name		NoELS-Vehicle	NoELS-URB	ELS-Vehicle	ELS-URB
	M: RQ	1	0.71	0.59	1.06
nfκb1	SEM	0.17	0.07	0.07	0.08
	F: RQ	1	1.3	0.87	0.87
	SEM	0.19	0.16	0.09	0.14
	M: RQ	1	1.09	0.88	1.19
il1β	SEM	0.12	0.04	0.04	0.1
	F: RQ	1	2	2.21	0.99
	SEM	0.26	0.21	0.15	0.12
	M: RQ	1	0.91	1	0.92
il6	SEM	0.09	0.07	0.06	0.1
	F: RQ	1	0.93	0.62	1.1
	SEM	0.12	0.24	0.07	0.11
	M: RQ	1	0.99	0.78	0.99
tnfα	SEM	0.09	0.1	0.06	0.15
	F: RQ	1	1.44	0.94	0.91
	SEM	0.09	0.19	0.12	0.1

ELS: early life stress; F: females; M: males; mPFC: medial prefrontal cortex; RQ: relative quantification; SEM: standard error of the mean; URB: URB597.

Table S4. Chronic late-adolescence URB597 treatment ameliorates ELS-induced changes in inflammatory gene expression in the mPFC.

		ELS[1]	ELS+URB[2]
nfκb1	M	↓	=
	F	↓	−
il1β	M	−	−
	F	↑	=
il6	M	−	−
	F	↓	=
tnfα	M	−	−
	F	↓	−

ELS: early life stress; F: females; M: males; URB: URB597.

[1] compared to NoELS-Vehicle.

[2] compared to ELS-Vehicle.

↑ Upregulation

↓ Downregulation

− No change

= Normalization

Table S5. Correlations between the expression of inflammatory genes in the mPFC and behavioral responses in adult males

	OFT- distance traveled	SP	SR	FST- swimming	FST- immobility
**nfκb1**	r = 0.221<br>p = 0.195	r = -0.22<br>p = 0.243	r = 0.37<br>p = 0.053	r = 0.251<br>p = 0.225	r = -0.153<br>p = 0.465
**il1β**	r = 0.25<br>p = 0.161	r = 0.317<br>p = 0.088	r = 0.143<br>p = 0.497	r = 0.427<br>p = 0.03	r = -0.2<br>p = 0.328
**il6**	r = 0.07<br>p = 0.707	r = -0.014<br>p = 0.945	r = 0.234<br>p = 0.25	r = -0.044<br>p = 0.839	r = -0.146<br>p = 0.497
**tnfα**	r = 0.13<br>p = 0.449	r = -0.053<br>p = 0.775	r = 0.181<br>p = 0.33	r = 0.356<br>p = 0.063	r = -0.236<br>p = 0.227

FST: forced swim test; OFT: open field test; SP: social preference; SR: social recognition.

Table S6. Correlations between the expression of inflammatory genes in the mPFC and behavioral response in adult females

	OFT- distance traveled	SP	SR	FST- swimming	FST- immobility
**nfκb1**	r = -0.089<br>p = 0.654	r = 0.176<br>p = 0.32	r = -0.18<br>p = 0.369	r = -0.407<br>p = 0.032	r = 0.464<br>p = 0.013
**il1β**	r = -0.349<br>p = 0.121	r = -0.359<br>p = 0.056	r = -0.016<br>p = 0.945	r = -0.298<br>p = 0.19	r = 0.522<br>p = 0.015
**il6**	r = 0.187<br>p = 0.403	r = 0.018<br>p = 0.93	r = -0.211<br>p = 0.359	r = 0.396<br>p = 0.068	r = -0.288<br>p = 0.194
**tnfα**	r = -0.222<br>p = 0.286	r = 0.051<br>p = 0.786	r = -0.289<br>p = 0.153	r = -0.434<br>p = 0.024	r = 0.382<br>p = 0.049

FST: forced swim test; OFT: open field test; SP: social preference; SR: social recognition.

Table S7. Descriptive statistics of stress-related gene expression in the mPFC.

Gene name		NoELS-Vehicle	NoELS-URB	ELS-Vehicle	ELS-URB
nr3c1	M: RQ	1	0.94	0.93	0.97
	SEM	0.07	0.06	0.06	0.04
	F: RQ	1	1.2	1.12	1
	SEM	0.13	0.14	0.1	0.14
crf	M: RQ	1	0.98	0.81	0.85
	SEM	0.01	0.007	0.005	0.005
	F: RQ	1	1.09	1.68	1.08
	SEM	0.12	0.15	0.23	0.15

crf: corticotropin releasing factor; ELS: early life stress; F: females; M: males; mPFC: medial prefrontal cortex; RQ: relative quantification; SEM: standard error of the mean; URB: URB597.

Table S8. Correlations between the expression of stress-related genes in the mPFC and behavioral responses in adult males

	**OFT- distance traveled**	**SP**	**SR**	**FST- swimming**	**FST- immobility**
**nr3c1**	r = 0.154<br>p = 0.408	r = 0.041<br>p = 0.834	r = -0.266<br>p = 0.231	r = 0.023<br>p = 0.922	r = -0.16<br>p = 0.487
**crf**	r = 0.348<br>p = 0.07	r = -0.093<br>p = 0.652	r = -0.006<br>p = 0.977	r = 0.131<br>p = 0.55	r = -0.284<br>p = 0.189

FST: forced swim test; OFT: open field test; SP: social preference; SR: social recognition.

Table S9. Correlations between the expression of stress-related genes in the mPFC and behavioral response in adult females

	**OFT- distance traveled**	**SP**	**SR**	**FST- swimming**	**FST- immobility**
**nr3c1**	r = -0.243<br>p = 0.222	r = 0.055<br>p = 0.759	r = -0.188<br>p = 0.339	r = -0.258<br>p = 0.185	r = 0.372<br>p = 0.05
**crf**	r = -0.301<br>p = 0.112	r = -0.225<br>p = 0.194	r = 0.127<br>p = 0.52	r = -0.105<br>p = 0.588	r = 0.229<br>p = 0.233

FST: forced swim test; OFT: open field test; SP: social preference; SR: social recognition.

Table S10. Descriptive statistics of inflammatory gene expression in the hippocampal CA1.

Gene name		NoELS-Vehicle	NoELS-URB	ELS-Vehicle	ELS-URB
nfκb1	M: RQ	1	0.99	1.12	0.75
	SEM	0.08	0.1	0.07	0.07
	F: RQ	1	0.93	1.02	1.19
	SEM	0.1	0.1	0.1	0.1
il1β	M: RQ	1	1.5	0.82	0.96
	SEM	0.07	0.11	0.11	0.07
	F: RQ	1	1.16	1.59	1.03
	SEM	0.12	0.25	0.16	0.08
il6	M: RQ	1	0.77	0.76	0.91
	SEM	0.09	0.05	0.05	0.03
	F: RQ	1	1.17	0.82	1.22
	SEM	0.11	0.24	0.11	0.09
tnfα	M: RQ	1	0.87	0.73	0.73
	SEM	0.13	0.1	0.05	0.05
	F: RQ	1	1.39	1.13	1.14
	SEM	0.11	0.17	0.12	0.19

ELS: early life stress; F: females; M: males; RQ: relative quantification; SEM: standard error of the mean; URB: URB597.

Table S11. Chronic late-adolescence URB597 treatment ameliorates ELS-induced changes in inflammatory gene expression in the hippocampal CA1.

		ELS[1]	ELS+URB[2]
nfκb1	M	—	↓
	F	—	—
il1β	M	↓	—
	F	↑	=
il6	M	—	—
	F	—	↑
tnfα	M	↓	—
	F	—	—

ELS: early life stress; F: females; M: males; URB: URB597.

[1] compared to NoELS-Vehicle.

[2] compared to ELS-Vehicle.

↑ Upregulation

↓ Downregulation

— No change

= Normalization

Table S12. Correlations between the expression of inflammatory genes in the hippocampal CA1 and behavioral response in adult males

	OFT- distance traveled	SP	SR	FST-swimming	FST-immobility
**nfκb1**	r = 0.126<br>p = 0.478	r = -0.238<br>p = 0.189	r = -0.237<br>p = 0.224	r = 0.165<br>p = 0.401	r = 0.071<br>p = 0.718
**il1β**	r = -0.043<br>p = 0.863	r = 0.091<br>p = 0.738	r = 0.078<br>p = 0.757	r = 0.075<br>p = 0.774	r = -0.077<br>p = 0.769
**il6**	r = 0.105<br>p = 0.642	r = 0.16<br>p = 0.455	r = 0.389<br>p = 0.09	r = 0.438<br>p = 0.061	r = -0.372<br>p = 0.117
**tnfα**	r = 0.117<br>p = 0.504	r = -0.177<br>p = 0.324	r = -0.141<br>p = 0.475	r = 0.324<br>p = 0.092	r = -0.395<br>p = 0.038

FST: forced swim test; OFT: open field test; SP: social preference; SR: social recognition.

Table S13. Correlations between the expression of inflammatory genes in hippocampal CA1 and behavioral responses in adult females

	OFT- distance traveled	SP	SR	FST-swimming	FST-immobility
**nfκb1**	r = 0.491<br>p = 0.009	r = -0.023<br>p = 0.9	r = -0.38<br>p = 0.05	r = 0.076<br>p = 0.711	r = -0.208<br>p = 0.307
**il1β**	r = -0.261<br>p = 0.312	r = -0.484<br>p = 0.026	r = -0.147<br>p = 0.548	r = -0.074<br>p = 0.757	r = -0.048<br>p = 0.842
**il6**	r = 0.105<br>p = 0.61	r = -0.272<br>p = 0.139	r = 0.238<br>p = 0.242	r = 0.183<br>p = 0.392	r = -0.255<br>p = 0.229
**tnfα**	r = 0.023<br>p = 0.912	r = -0.283<br>p = 0.117	r = -0.093<br>p = 0.637	r = -0.052<br>p = 0.805	r = -0.015<br>p = 0.942

FST: forced swim test; OFT: open field test; SP: social preference; SR: social recognition.
